# Molecular Cloning, Promoter Analysis and Expression Profiles of the *sox3* Gene in Japanese Flounder, *Paralichthys olivaceus*

**DOI:** 10.3390/ijms161126079

**Published:** 2015-11-24

**Authors:** Jinning Gao, Peizhen Li, Wei Zhang, Zhigang Wang, Xubo Wang, Quanqi Zhang

**Affiliations:** 1 Center for Developmental Cardiology, Institute for Translational Medicine, College of Medicine, Qingdao University, 38 Dengzhou Road, Qingdao 266021, China; gjn.1127@163.com; 2 Key Laboratory of Marine Genetics and Breeding (MGB), Ministry of Education, College of Marine Life Sciences, Ocean University of China, 5 Yushan Road, Qingdao 266003, China; peizhenlee@163.com (P.L.); 18366207357@163.com (W.Z.); zgwang@ouc.edu.cn (Z.W.)

**Keywords:** Japanese flounder (*Paralichthys olivaceus*), *sox3*, promoter analysis, sexually dimorphic expression

## Abstract

Sox3, which belongs to the SoxB1 subgroup, plays major roles in neural and gonadal development. In the present study, Japanese flounder *Paralichthys olivaceus sox3* gene (*Posox3*) and its promoter sequence were isolated and characterized. The deduced PoSox3 protein contained 298 amino acids with a characteristic HMG-box domain. Alignment and phylogenetic analyses indicated that PoSox3 shares highly identical sequence with Sox3 homologues from different species. The promoter region of *Posox3* has many potential transcription factor (TF) binding sites. The expression profiles of *Posox3* in different developmental stages and diverse adult tissues were analyzed by quantitative real-time RT-PCR (qRT-PCR). *Posox3* mRNA was maternally inherited, and maintained at a considerably high expression level between the blastula stage and the hatching stage during embryonic development. *Posox3* was abundantly expressed in the adult brain and showed sexually dimorphic expression pattern. *In situ* hybridization (ISH) was carried out to investigate the cellular distribution of *Posox3* in the ovary, and results showed the uniform distribution of *Posox3* throughout the cytoplasm of oogonia and stage I–III oocytes. These results indicate that *Posox3* has potentially vital roles in embryonic and neural development and may be involved in the oogenesis process. Our work provides a fundamental understanding of the structure and potential functions of Sox3 in *Paralichthys olivaceus*.

## 1. Introduction

The Sox protein family, which participates in diverse developmental events, has been classified into group A–J based on the HMG domain phylogeny [1]. The molecules in the SoxB1 subgroup, including Sox1, Sox2 and Sox3, share more than 90% amino acid identity in their characteristic HMG box domains. SoxB1 proteins are indicated to have overlapping properties and tend to function interactively and redundantly [2,3,4]. Significantly, *SoxB1* genes continue to be expressed in the developing central nervous system (CNS) and can maintain the neural progenitor identity [5,6,7,8]. Nevertheless, their functions are not strictly redundant and each of them possesses distinct roles during embryonic development and in cell fate determination. Among them, Sox2 is well known as a pluripotency factor that cooperates with Oct4 and Nanog in regulating the pluripotent state of embryonic stem cells [9,10]. Sox1 appears to play a direct role in promoting neural determination and differentiation [11]. Sox3, which is considered to be the ancestral precursor of *Sry*, is required for sex differentiation and gonadal development [12,13].

Fish are the most diverse and species-rich group of vertebrates, serving as an evolutionary link between invertebrate and higher vertebrates. So far, few studies have been reported on the teleost *sox3* gene. Diverse expression profiles of *soxB1* during early embryo development have been observed in zebrafish when compared with those in amniotes, but their overall functions in neural development may be conserved among vertebrate species [14]. Specifically, gain- and loss-of-function experiments in zebrafish elucidate the role of Sox3 both in neural fate determination and differentiation [15]. In grouper *Epinephelus coioides*, dynamic expression pattern of Sox3 in the gonad confirms its potential role in oogenesis and germ cell differentiation [16]. In catfish *Clarias batrachus*, results suggest a possible role for Sox3 in the regulation of testicular development [17]. Recently, Takehana, *et al.* [18] have shown that *Sox3* initiates testicular differentiation in *Oryzias dancena*.

A promoter is a vital region containing various elements and TF binding sites that can regulate gene expression. For the past ten years, Milena Stevanovic′s team concentrated on the functional characterization of the human *SOX3* promoter [19]; they have conducted experiments to demonstrate that TFs, such as NF-Y, TGIF, PBX1 and MEIS1, and CREB, are key regulators of *SOX3* expression, either in a positive or negative way [20,21,22,23]. Also, it has been reported that a small ubiquitin-like modifier (SUMO) represses transcriptional activity of *SOX3* at the posttranscriptional level [24]. However, little is known about the transcriptional regulation of *sox3* in lower vertebrate, especially in the marine fish.

The Japanese flounder, *Paralichthys olivaceus*, is widely distributed along the coastal shelf of Northeast Asia and is one of the most promising and commercially important flatfish species in the region. Investigation on the *soxB1* genes is of interest and significant benefit for further study. We have previously isolated and characterized the *sox1* and *sox2* genes in *P. olivaceus*, which are predominately expressed at the early development stages and in the brain tissue [25,26]. Moreover, we demonstrated that two co-orthologs of *sox1* (*Posox1a* and *Posox1b*) exist in the flounder genome generated by gene duplication. However, the expression of *Posox2* may not be regulated through the CpG sites in its regulatory region. Thus, elucidating the role of *Posox3* is necessary to form a comprehensive understanding of *soxB1* genes in *P. olivaceus*. For these purposes, here we describe the molecular characterization of Japanese flounder *sox3*, its quantified expression profiles during early embryo development and in diverse adult tissues, and analyses of its cellular distribution in the ovary. In addition, analysis of potential regulatory motifs in the upstream region was conducted to facilitate further functional studies in this species.

## 2. Results

### 2.1. Molecular Characterization of Posox3

#### 2.1.1. Cloning and Sequence Analysis of *Posox3*

**Figure 1:**
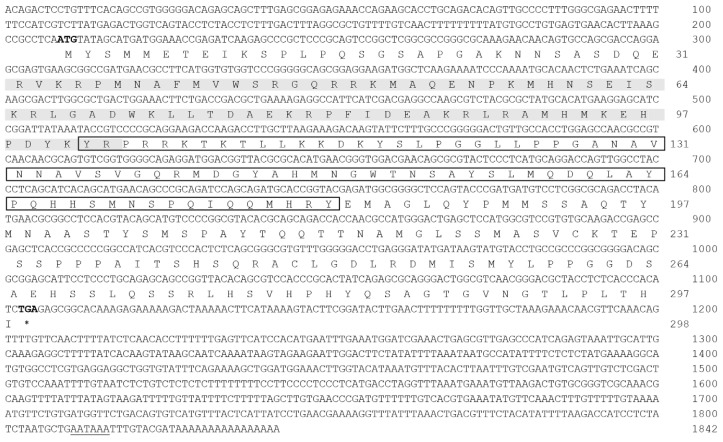
The nucleotide and deduced amino acid sequences of *Posox3*. The HMG (high mobility group) box domain is shaded in gray and the SOXp (SOX transcription factor) domain is boxed. The start and stop codons are bold-typed and the stop codon is represented as asterisk (*). The polyadenylation signal (AATAAA) is indicated with single line. Nucleotides and amino acids are numbered at the right end of the lines.

Using degenerate primers and RACE strategy, we cloned *Posox3* from the brain tissue of Japanese flounder. All obtained fragments were assembled to yield a cDNA with 208 bp of 5′ UTR, 737 bp of 3′ UTR and 897 bp of the entire open reading frame (ORF). A putative polyadenylation signal (ATTAAA) was found 10 bp upstream of the poly (A) tail (GenBank accession number: KR108248) (Figure 1). The predicted PoSox3 amino acid sequence was 298 amino acids with an estimated molecular mass (MM) of 33.22 kDa and an estimated isoelectric point (IP) of 9.66. Similarly with other Sox proteins, PoSox3 had the characteristic HMG-box DNA binding domain (72 aa), located on the amino acids 32 to 103, and the SOX transcription factor (SOXp) domain (80 aa) at 102–181 aa (Figure 1).

#### 2.1.2. Protein Alignment and Phylogenetic Analysis of PoSox3

**Figure 2:**
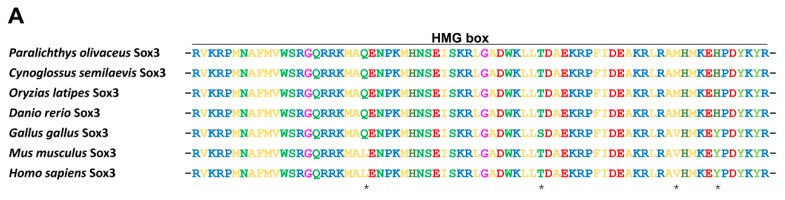

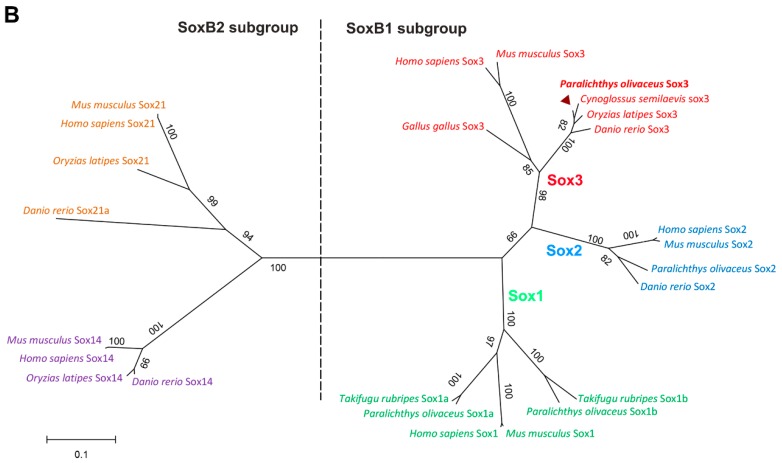
PoSox3 amino acids alignment and phylogeny. (**A**) Alignment of HMG-box domain of PoSox3 with those of other vertebrate orthologues. Different amino acid residues between fish and tetrapods are indicated with asterisks; (**B**) Phylogenetic relationships of PoSox3 and SoxB proteins from other representative species. The Neighbor-Joining tree was constructed by MEGA (version 6.06) with 1000 bootstrapping based on the full-length amino acids sequences. The sequences were obtained with the following GenBank accession numbers: *Homo sapiens*: NP_005977.2 for Sox1; NP_003097.1 for Sox2; NP_005625.2 for Sox3; NP_004180.1 for Sox14; NP_009015.1 for Sox21; *Mus musculus*: NP_033259.2 for Sox1; NP_035573.3 for Sox2; NP_033263.2 for Sox3; NP_035570.1 for Sox14; NP_808421.1 for Sox21; *Gallus gallus*: NP_989526.1 for Sox3; *Danio rerio*: NP_998283.1 for Sox2; NP_001001811.2 for Sox3; NP_001032769.1 for Sox14; NP_571361.1 for Sox21a; *Oryzias latipes*: NP_001098234.1 for Sox3; NP_001158344.1 for Sox14; NP_001158346.1 for Sox21; *Cynoglossus semilaevis*: XP_008324981.1 for Sox3; *Paralichthys olivaceus*: KR108250 for Sox1a; KR108247 for Sox1b; KF709692 for Sox2; KR108248 for Sox3 (solid triangle); *Takifugu rubripes*: XP_003961796.1 for Sox1a; XP_003977952.1 for Sox1b. Distinct clades were indicated with different colors.

The amino acid sequence of PoSox3 was compared to known Sox3 proteins from other ray-finned fish and higher vertebrates. The overall identities between PoSox3 and other Sox3 were extremely high, from 75.5% with *H. sapiens* Sox3 to 97.7% with *X. maculatus* Sox3 (Figure S1). The alignment confirmed the presence of the conserved HMG-box domain within PoSox3, which is the characteristic of Sox proteins (Figure 2A). To gain insight of the evolutionary relationship between PoSox3 identified in this study and the SoxB molecules of other vertebrate, a phylogenetic tree was constructed (Figure 2B), which showed two distinct clades representing SoxB1 and SoxB2 subgroups. The clade of SoxB1 consisted of three subclades, namely Sox1, Sox2 subclades and a Sox3 subclade containing PoSox3. PoSox3 then clustered with its homologues in teleost. Moreover, the molecular relationship indicated by this tree was consistent with the taxonomic classification of these species.

#### 2.1.3. Genomic Organization and Analysis of *Posox3*

**Figure 3:**
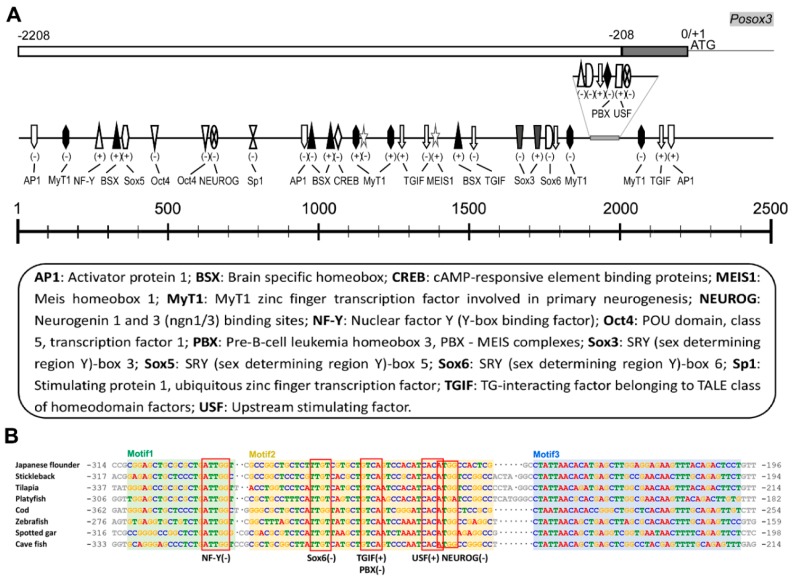
Bioinformatic analyses of the promoter sequence of *Posox3*. (**A**) The potential TF binding sites were drawn on the schematic diagram with their corresponding locations within the promoter of *Posox3*. The scale is shown above and the detailed information are supplied at the bottom. The plus-minus symbols indicate the strand with which the TFs bind. The gray block represents the conserved upstream region among different fish species. The details for TFs see in Table S1; (**B**) Consensus motif regions in the 5′ upstream sequences of different fish *sox3* orthologs analyzed by software MEME and Dialign. The GenBank accession number of Japanese flounder (*Paralichthys olivaceus*) *sox3* was KT314157. Other sox3 5′-flanking sequences were searched from Ensembl [28] and their gene IDs were as follows: Stickleback (*Gasterosteus aculeatus*) *sox3*: ENSGACG00000017181; Tilapia (*Oreochromis niloticus*) *sox3*: ENSONIG00000020861; Platyfish (*Xiphophorus maculatus*) *sox3*: ENSXMAG00000019515; Cod (*Gadus morhua*) *sox3*: ENSGMOG00000020190; Zebrafish (*Danio rerio*) *sox3*: ENSDARG00000053569; Spotted gar (*Lepisosteus oculatus*) *sox3*: ENSLOCG00000017493; Cave fish (*Astyanax mexicanus*) *sox3*: ENSAMXG00000025368. The potential TF (NF-Y, Sox6, TGIF/PBX, USF, and NEUROG) binding sites within the conserved motifs are boxed and indicated above the aligned sequences.

Comparison of the genomic (GenBank accession number: KT314157) and cDNA sequences showed that *Posox3* was a one-exon gene. Numerous putative binding sites for TFs involved in cellular proliferation and differentiation as well as ubiquitous TFs were revealed by online software MatInspector in the ~2.2 kb region upstream of the start codon (Figure 3A, Table S1). Several Sox/sry-sex/testis-determining and related HMG box factors, including Sox5, Sox6, and Sox3 itself, were identified. Some of these TFs, such as brain specific homebox (BSX), neurogenin 1 and 3 (NEUROG), TG-interacting homeodomain factor (TGIF), and myelin transcription factor 1 (MyT1), were involved in neurogenesis. Furthermore, other development related factors including POU domain, class 5, transcription factor1 (Oct4), pre-B-cell leukemia homeobox 3 (PBX), myeloid ecotropic viral integration site 1 homologue (MEIS1) and cAMP-responsive element binding protein (CREB) were identified. Ubiquitous binding sites for activator protein 1 (AP1), nuclear factor Y (NF-Y), stimulating protein 1 (Sp1), and upstream stimulating factor (USF) were also found. Unlike the mammalian *Sox3* [27], the canonical TATA box was not found in the promoter region of *Posox3*. Nevertheless, at least 100 bp-long evolutionary conserved regulatory region was revealed by the comparative genomic analysis of fish *sox3* orthologous promoters (Figure 3B).

### 2.2. Expression Profiles of Posox3 during Embryonic Development and in the Larval and Juveniles

**Figure 4:**
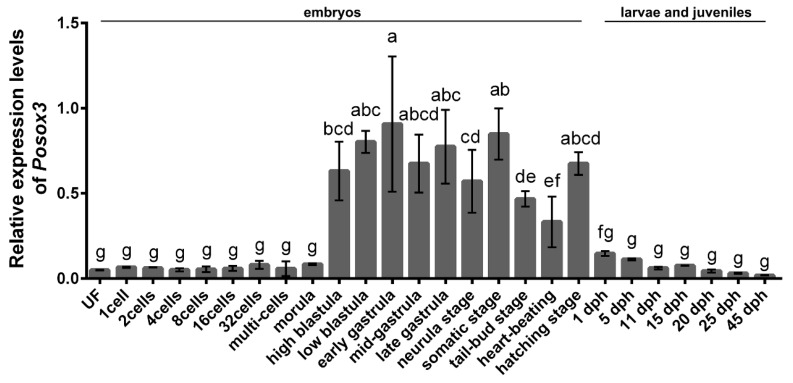
qRT-PCR analysis of *Posox*3 during embryonic development and in larvae and juveniles. Relative mRNA level is shown as mean ± SEM (*n* = 3). The relative expression variance is given as ratio (the amounts of *Posox*3 mRNA/reference genes). Different letters indicate significant difference (*p* < 0.05). UN, unfertilized egg; dph, days post-hatching.

To evaluate the temporal expression levels of *Posox3* during early embryo development as well as in the larvae and juveniles, we conducted qRT-PCR analysis. *Posox3* mRNA could already be detected in the unfertilized egg. With the early embryonic cleavage and subsequent development proceeding, the amount of transcript remained stable at a low level until the morula stage, strongly increased at the high-blastula stage, then reached the peak at the early gastrula stage. The *Posox3* transcript was greatly diminished after hatching, and gradually decreased from 1 day post-hatching (dph) to 45 dph. In general, *Posox3* was highly expressed from the high-blastula stage to the hatching stage, and had low expression levels at the other examined stages (Figure 4).

### 2.3. Expression Profiles of Posox3 in Different Tissues and in Adult Brain

**Figure 5:**
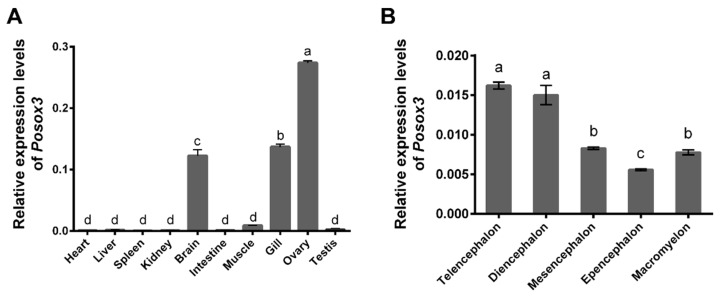
qRT-PCR analysis of *Posox*3 in different adult tissues (**A**) and in different parts of adult brain (**B**). Relative mRNA level is shown as mean ± SEM (*n* = 3). The relative expression variance is given as ratio (the amounts of *Posox*3 mRNA/reference genes). Different letters indicate significant difference (*p* < 0.05).

To examine the tissue distribution of *Posox3*, we performed qRT-PCR analysis using RNA from various tissues of 1.5-year-old adult fish. The results showed that *Posox3* was highly expressed in ovary, gill and brain, low in muscle and testis, and almost undetectable in heart, liver, spleen, kidney and intestine tissues (Figure 5A). The expression of *sox3* in gill tissue in addition to gonads and brain has also been found in other fish species [18]. Notably, *Posox3* showed a sexually dimorphic expression pattern that the transcript was abundant in ovary but deficient in testis.

The *Posox3* transcript was highly expressed in the brain, suggesting its potential role in neural development. To acquire further information, we then analyzed its expression profiles among five different parts of the whole brain. Telencephalon and diencephalon showed high expression levels of the *Posox3* mRNA transcript, while decreasing levels were observed in the mesencephalon, macromyelon, and epencephalon (Figure 5B).

### 2.4. In Situ Hybridization

**Figure 6:**
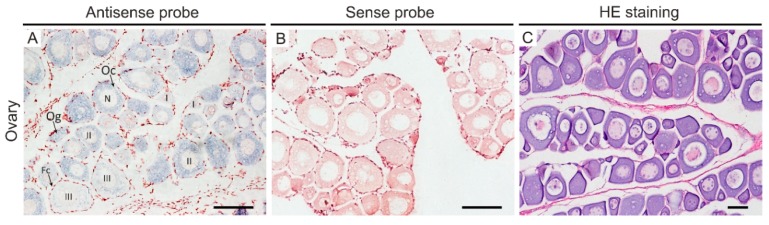
Spatiotemporal expression pattern of *Posox3* mRNA in the ovary of adult Japanese flounder. *In situ* hybridization was performed on paraffin-embedded tissues with a DIG-labeled *Posox3* antisense probe (**A**) or a sense probe as a control (**B**). The positive signals are stained with blue, whereas the negative control is not stained. HE staining in the ovary (**C**), counterpart to A. In ovary, the *Posox3* mRNA is widely distributed in the cytoplasm of different stages oocytes and highly expressed in stage II oocytes. Fc, follicle cells; N, nucleus; Oc, oocytes; Og, oogonia; I–III, oocyte stages. Scale bar, 100 µm.

To elucidate localization of *Posox3* mRNA at the cellular level, we carried out *in situ* hybridization on paraffin-embedded ovary sections of Japanese flounder. The ovary section for ISH is composed of a small amount of oogonia, numbers of oocytes at different development stages including stage I (small), stage II (previtellogenic) and stage III (vitellogenic), as well as somatic follicle cells (Figure 6C). ISH revealed that *Posox3* RNA was exclusively restricted to the germ cells, and no signal was detected in the surrounding somatic follicle cells. Moreover, *Posox3* RNA was expressed throughout oogenesis and showed uniform distribution throughout the cytoplasm of oogonia and stage I–III oocytes (Figure 6A). However, no signal was observed in the control experiment using sense probes of *Posox3* (Figure 6B).

## 3. Discussion

### 3.1. Posox3 Is the Homolog of Mammalian Sox3

In this study, we isolated and characterized the complete sequence of *sox3* as well as its promoter region from a flatfish, Japanese flounder *P. olivaceus*. We conclude that *Posox3* encodes the Japanese flounder Sox3 and is homologous to the mammalian *Sox3* for the following reasons. Firstly, *Posox3* is a one-exon gene. This is coincident with other reported *SOX3*/*Sox3* genes [29,30]. Moreover, along with our previously studies [25,26], *P. olivaceus soxB1* genes (*Posox1a/b*, *Posox2*, and *Posox3*) are all composed of a single exon, which is the conserved characteristic of the vertebrate *SoxB1* subgroup [1]. Secondly, the HMG-box domain of PoSox3 contained the nine specificity amino acids, RPMNAFWVW (positions, 4–12), which appears to be the most reliable signature of the Sox family [1]. Further, alignment of the predicted PoSox3 with other Sox3 proteins from various species shows overall high identity (75.5%–97.7%), especially within the conserved HMG-box domain and its C-proximal region. These suggest that the Sox3 protein may be structurally conserved during vertebrate evolution. Finally, on a phylogenetic tree, the putative PoSox3 is firstly clustered with other fish Sox3 proteins, coinciding with the separation between fish and tetrapod linages, then clustered with Sox2 and Sox1 proteins to form the SoxB1 clade. Taken together, these results indicate that the described *Posox3* is the ortholog of mammalian *Sox3*.

### 3.2. The Potential TFs in Regulating Posox3 Expression

Given the recognized importance of *Sox3* in the regulation of embryonic development and in the determination of cell fate, characterization of the promoter sequence and identification of the molecular mechanism will contribute a better understanding to the regulation of its expression and function. Here, we analyze the 2000 bp 5′ upstream sequence of Japanese flounder *sox3* through bioinformatics software to identify a number of putative TF binding sites. The TFs, including Sox3, Sox5, Sox6, Oct4, BSX, NEUROG, MyT1, TGIF, CREB, PBX, MEIS1, NF-Y, Sp1, AP1 and USF, may be involved in the regulation of *Posox3* expression and function.

Many transcription factors, including Sox factors, have been reported to have autoregulatory regions, where the protein can directly bind to or mediate with specific partners to achieve autoregulation [31,32,33]. Further, ChIP-seq analyses have indicated that *Sox3* is one of the small subset of SOX3 direct target genes which require SOX3 for normal expression in the neural progenitors [34]. Interestingly, two binding sites for Sox3 itself are found in the *Posox3* promoter region, suggesting the potential mechanism of autoregulation.

Different from the consensus co-factors-binding site for PBX/MEIS, which has been demonstrated to participate in positively regulating human *SOX3* gene expression by direct interaction [23], we identified separately located PBX and MEIS1 in the promoter region of *Posox3*. This is not surprising, for we have found binding sites for Oct4 in the Japanese flounder *Nanog* gene promoter, but not the mammalian composite Oct-Sox element in our previous study [35]. Moreover, heterologous DNA often functions similarly in organisms even if they share little regulatory sequence similarities (e.g., human DNA in fish) [36]. This may suggest regulatory sequence evolution and potential conservative functions from fish to mammals.

Oct4, a POU domain transcription factor, is critically involved in the stem cell self-renewal and pluripotency maintenance. It may interact with Sox3 to keep neural cells undifferentiated as Sox3 is found expressed in self-renewing neural progenitors [7,34,37]. Ubiquitous TFs, such as USF, Sp1 and NF-Y, which play important roles in diverse cellular processes, have been demonstrated to regulate the expression of several members of *Sox* gene family [19,20,38]. Our first step of promoter analysis was based on computational prediction, and whether these factors play conserved roles in regulating *Posox3* expression needs verification. Still, the current results will provide fundamental information for further investigation on the transcriptional regulation mechanism and functional analysis of *Posox3*.

### 3.3. Posox3 May Be Involved in the Regulation of Embryogenesis, Neural and Ovarian Development

So far, few studies have reported the temporal expression profiles of *sox3* during early embryogenesis in fish. The embryonic expression of *Posox3* RNA is in general coincident with those that have been observed in red-spotted grouper and zebrafish, but different from annual killfish [14,39,40]. In our study, *Posox3* is maternally inherited, sustained at a low level until morula stage, then sharply increases from blastula stage on when zygotic transcription begins, and this high expression level persists until the hatching stage. Minor difference exists after hatching, and the transcript declines dramatically to a low level in Japanese flounder but is still highly expressed in red-spotted grouper [39]. Differently from *Posox1a/b* and *Posox2*, which are highly expressed at a certain stage (the neurula stage and the gastrula stage, respectively) [25,26], the *Posox3* transcript is abundant for a period of time during embryogenesis. It is important that germ layer differentiation, histogenesis and organogenesis occur during embryonic development. Our observation supports the speculation that a steady level of *Posox3* is associated with embryogenesis.

Sox3 is critical for neural development in vertebrates [4,14,41,42], and Sox3 deficient mice have defects in CNS midline structures [43]. In zebrafish, knockdown of Sox3 reduces the size of the CNS and subsequently inhibits some aspects of neurogenesis [15]. In addition, Sox3 plays an important role in maintaining neural progenitors residing in the telencephalic stem cell niches [7,8,44]. Tissue distribution analysis shows that *Posox3* is highly expressed in brain tissue, especially abundantly in the telencephalon region, which suggests its potential vital role in the CNS.

In addition to the neural tissue, Sox3 is also expressed in the gonadal tissue. In mouse, Sox3 has been demonstrated to be important for oocyte development, testis differentiation as well as gametogenesis [12]. However, in species-rich fish, diverse expression profiles reveal their distinct roles, mainly functioning either in testicular or in ovarian development. In protogynous hermaphrodite fish *Epinephelus coioides*, *Ecsox3* has been suggested to have a more important role in oogenesis than in spermatogenesis, whereas *sox3* is expressed highly in testis and involved in the development of testis more than ovary in protandrous hermaphrodite fish *Acanthopagrus schlegeli* [16,45]. In *Clarias batrachus*, *sox3* shows a possible role in the regulation of testicular development [17]. In our present study, *Posox3* is more highly expressed in the ovary than in the testis. This expression pattern is in agreement with what has been observed in red-spotted grouper, but in contrast to black porgy and catfish [16,17,45]. Thus, the sexually dimorphic expression pattern of *Posox3* suggests it may play a more important role in ovary than testis in *P. olivaceus*.

## 4. Experimental Section

### 4.1. Animals and Sampling

*P. olivaceus* (*Pleuronectiformes*) individuals used in this study were collected in a commercial fish farm in Haiyang, China. Animal experiments were performed according to the Regulations for the Administration of Affairs Concerning Experimental Animals (China, 1988). The procedures were also approved by College of Marine Life, Ocean University of China (Qingdao, China). The embryos were observed under a dissecting microscope and divided into different stages. Embryos of certain stages, including unfertilized eggs (UF), 1 cell, 2 cells, 4 cells, 8 cells, 16 cells, 32 cells, multi-cells, morula, high blastula, low blastula, early gastrula, mid-gastrula, late gastrula, neurula, somatic, tail-bud, heart-beating, and hatching stages, and seven larvae and juvenile stages at 1, 5, 11, 15, 20, 25, and 46 days post-hatching (dph), were collected in triplicate using 100 μm mesh nylon screen, washed with phosphate buffered saline (PBS) and stored in RNAwait (Solarbio, Beijing, China) for further experiment. Adult individuals were acclimatized in laboratory environment for 48 h before the sampling, after which six individuals (three males and three females) were randomly selected for the tissue sampling of heart, liver, spleen, kidney, whole brain, intestine, gonads, gill and skeletal muscles. The tissue samples were quick-frozen in liquid nitrogen and then stored at −80 °C for further experiment.

### 4.2. Total RNA and Genomic DNA Extraction

Total RNA were extracted from multiple tissues or different developmental stages using TRIzol reagent (Invitrogen, Carlsbad, CA, USA) according to the manufacturer’s instructions. DNA contamination was detected by β*-actin* primers (spanning different exons, Table 1, β-actin-Fw/Rv) and eliminated by DNase I (TaKaRa, Dalian, China). Genomic DNA was extracted from muscle tissues with the phenol-chloroform method. All the extracted RNA and DNA samples were examined by agarose gel electrophoresis and quantified using Nanophotometer Pearl (Implen GmbH, Munich, Germany).

**Table 1:** Sequences of the primers used in this study.

Experiment	Primer Name	Primer Sequences (5′–3′)
DNA detection	β*-actin-Fw*	GAGATGAAGCCCAGAGCAAGAG
	β*-actin-Rv*	CAGCTGTGGTGGTGAAGGAGTAG
Core fragment	*sox3-core-Fw*	AGCCGCCTCAATGTATAGCA
	*sox3-core-Rv*	GCGTAACCGTCCATCCTCT
5′ RACE PCR	*5′ RACE-sox3-467*	GTCGGTCAGAAGTTTCCAGTCAG
	*5′ RACE-sox3-327*	GGTCGCTGGCACTGTTGTTCT
3′ RACE PCR	*3′ RACE-sox3-654*	CAGAGGATGGACGGTTACGC
	*3′ RACE-sox3-827*	TACACGCAGCAGACCACCAAC
Full-length cDNA	*sox3-full-length-Fw*	AACATTGAACATGATTACGATTCG
	*sox3-full-length-Rv*	ATCGTACAAATTTATTCAGCATTAG
Promoter	*sox3-promoter-Fw*	GCCTGTATTTGTAGTCTAAT
	*sox3-promoter-Rv*	TGAGGCGGCTTTAAGTGTT
qRT-PCR	*sox3-RT-Fw*	AACAACGCAGTGTCGGTGG
	*sox3-RT-Rv*	TGCTGTGATGCTGAGGGTAGG
qRT-PCR	*18S-RT-Fw*	GGTAACGGGGAATCAGGGT
	*18S-RT-Rv*	TGCCTTCCTTGGATGTGGT
qRT-PCR	*UbcE-RT-Fw*	TTACTGTCCATTTCCCCACTGAC
	*UbcE-RT-Rv*	GACCACTGCGACCTCAAGATG
ISH	*sox3-ISH-SP6*	ATTTAGGTGACACTATAGAAGTG
		TACACGCAGCAGACCACCAAC
ISH	*sox3-ISH-T7*	TAATACGACTCACTATAGGGAGA
		AAATACACCAGCCTCCTCACG

### 4.3. Molecular Cloning of the Full Length cDNA and Promoter Sequence of Posox3

Total RNA of the brain tissues was used to obtain the full-length cDNA of *Posox3*. Briefly, the first strand cDNA was synthesized from 1 μg of RNA using M-MLV reverse transcriptase (RNase H-) (TaKaRa) and random primers according to the manufacturer’s instructions. A positive fragment (1100 bp) sharing high identities with *Epinephelus coioides sox3* and *Larimichthys crocea sox3* was found by searching the *de novo* transcriptome sequencing data of *P. olivaceus* [46]. Primer pair sox3-core-Fw/Rv was designed to verify the core fragment and ensure sequence accuracy. The remaining unknown UTRs were obtained by 5′ and 3′ RACE using the SMART™ RACE cDNA Amplification kit (Clontech, Carlsbad, CA, USA) according to manufacturer’s protocol, using the universal primer mix (UPM), gene-specific primers (5′ RACE-sox3-467 and 5′ RACE-sox3-327) for 5′-end sequence, and the nested universal primer (NUP), gene-specific primers (3′ RACE-sox3-654/827) for 3′-end sequence. Specific primers sox3-full-length-Fw/Rv, which flanked the entire ORF, were designed based on the assembled cDNA sequence to obtain the genomic DNA sequence and to confirm the full-length coding sequence.

Based on the *P. olivaceus* genomic data, degenerate primer pair sox3-promoter-Fw/Rv was designed to amplify and verify the 2000 bp 5′ upstream sequence of *Posox3*. DNA fragments that were in the range of expected PCR product size were excised with a razor blade, purified using the Zymoclean gel-purification kit (Zymo Research, Orange, CA, USA), cloned into a pMD 18-T vector (TaKaRa, Dalian, China) and sequenced.

### 4.4. Sequence Analysis

Analyses of the nucleotide and protein sequences were performed with software Lasergene v7.0 (DNASTAR, Madison, WI, USA) and the National Center for Biotechnology Information [47]. Multiple alignments of amino acid sequences were carried out using the ClustalW2 program [48], and the results were applied to the generation of a phylogenetic tree by MEGA 6.06. The conserved domains of deduced PoSox3 were analyzed using the simple modular architecture research tool (SMART) [49] and InterProScan search software [50]. The potential TF binding sites within the 5′ upstream region of *Posox3* were predicted via the online program MatInspector (Matrix Family Library Version 9.2) [51]. Comparison of the 5′-flanking regions of *sox3* among different fishes was performed with mVISTA using the alignment program AVID [52] and the MEME Suite 4.10.2 [53].

### 4.5. Quantitative Real-Time RT-PCR (qRT-PCR)

The cDNA templates for qRT-PCR assays were synthesized using the method described above. Pre-experiment was conducted to confirm the specific cDNA PCR product we tested. qRT-PCR was performed in a 20 μL reaction volume with cDNA templates (5 ng), the specific primers (sox3-RT-Fw/Rv, 0.2 μM), 1 × SYBR Premix Ex Taq™ II (Perfect Real Time, TaKaRa) using Light-Cycler Roche 480 (Roche Applied Science, Mannheim, Germany). Melting curves were generated following each cycle to confirm the specificity of the amplicons. Blank controls (with no template) were always included. The relative expression levels of target gene were calculated through a standard curve for quantitation as we previously described [35]. *18s rRNA* and *UbcE* were employed as reference genes [54,55]. All qRT-PCR assays were performed in triplicate under identical conditions. All primers were listed in Table 1.

### 4.6. In Situ Hybridization and Histological Analysis

For ISH analysis, ovary was fixed in 4% paraformaldehyde–PBS (4% PFA) overnight at 4 °C, dehydrated using graded methanol and stored in pure methanol at −20 °C. The same sample used for histological observation was fixed in Bouin’s fixative for 24 h and stored in 70% ethanol at 4 °C.

ISH analysis of *Posox3* expression in the ovary was carried out using a 594 bp probe (positions, 836–1429) containing partial 3′ UTR and the C-terminal coding region of *Posox3* (Table 1, sox3-ISH-SP6/T7). Digoxigenin (DIG)-labeled antisense and sense riboprobes were synthesized with DIG RNA Labeling Kit (SP6/T7) according to the manufacturer’s instructions (Roche). For histological analysis, sections were stained with hematoxylin and eosin (HE). In brief, ISH and HE staining on paraffin-embedded sections of ovary were performed as previously described [35,56]. The sections were observed and photographed by using a Nikon Eclipse Ti-U microscope (Nikon, Tokyo, Japan).

### 4.7. Statistical Analysis

All data were shown as mean ± standard error of the mean (SEM). Statistical analyses were performed using one-way analysis of variance (ANOVA) followed by Duncan’s test in SPSS 20.0 (IBM, New York, NY, USA). *p* < 0.05 indicated statistically significant differences among samples.

## 5. Conclusions

In summary, the present study provides the complete cDNA sequence of *sox3* gene in *P. olivaceus*. By gene structure, sequence comparison and phylogenetic analysis, *Posox3* is shown to be the homolog of mammalian *Sox3*. Several regulatory motifs are found in the 5′ upstream region, suggesting their potential roles in regulating gene expression and function. In addition, we have surveyed the expression patterns by qRT-PCR and ISH. *Posox3* expresses at a steady high level from the blastula stage to the hatching stage during early embryogenesis. *Posox3* is highly expressed in the adult brain and shows sexually dimorphic expression pattern. These results will provide fundamental information for further functional investigation of *sox3* in *Paralichthys olivaceus*.

## Supplementary Materials

Supplementary materials can be found at http://www.mdpi.com/1422-0067/16/11/26079/s1.
