# Supplementary material for: Molecular Cloning, Promoter Analysis and Expression Profiles of the sox3 Gene in Japanese Flounder, Paralichthys olivaceus
Source: Int J Mol Sci. 2015 Nov 24;16(11):27931–44. doi: 10.3390/ijms161126079 (PMC4661933; doi:10.3390/ijms161126079)
Supplement: Supplementary file 1 [file ijms-16-26079-s001.pdf]

Supplementary Materials: Molecular Cloning, Promoter Analysis and Expression Profiles of the *sox3* Gene in Japanese Flounder, *Paralichthys olivaceus*

Jinning Gao, Peizhen Li, Wei Zhang, Zhigang Wang, Xubo Wang and Quanqi Zhang

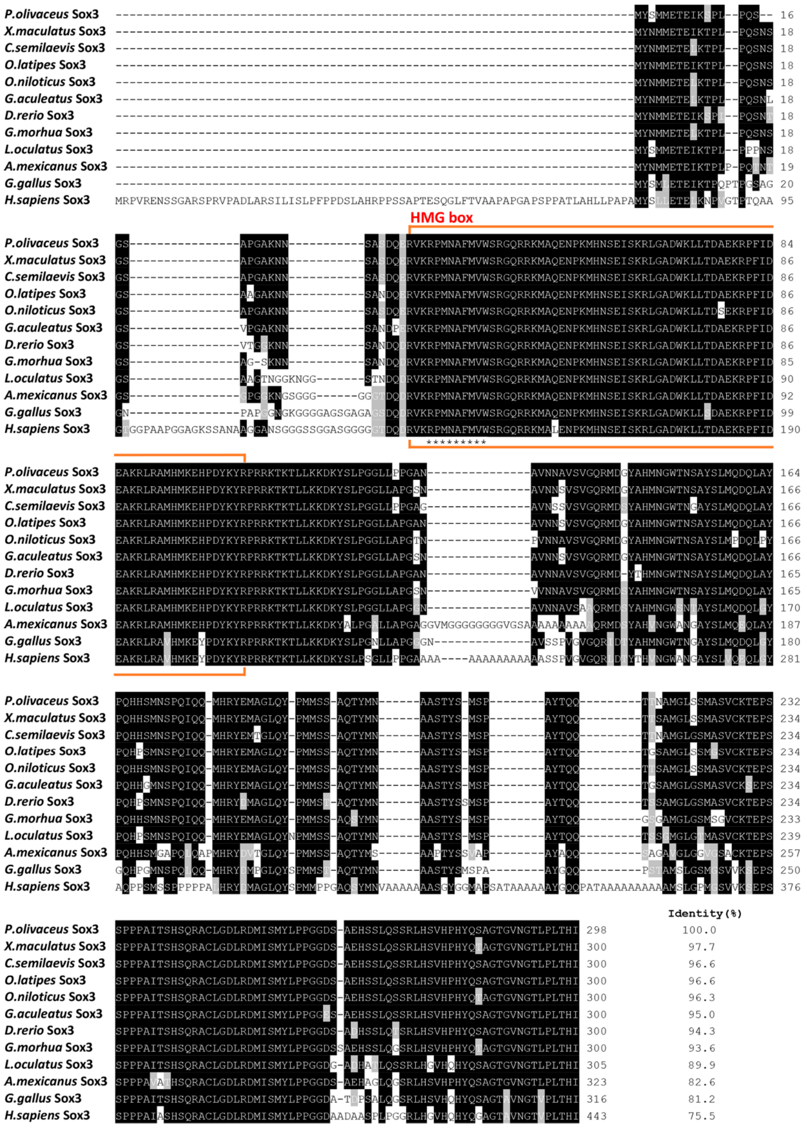

Figure S1. Multiple alignment of Sox3 proteins in different species. All the sequences of Sox3 homologues were retrieved from NCBI and the Ensemble database. The GenBank accession numbers or Ensembl IDs are as follows: *P. olivaceus* (*Paralichthys olivaceus*), KR108248; *X.maculatus* (*Xiphophorus maculatus*), ENSXMAG00000019515; *C.semilaevis* (*Cynoglossus semilaevis*), XP_008324981.1; *O.latipes* (*Oryzias latipes*), NP_001098234.1; *O.niloticus* (*Oreochromis niloticus*), ENSONIG00000020861; *G.aculeatus* (*Gasterosteus aculeatus*), ENSGACG00000017181; *D.rerio* (*Danio rerio*), NP_001001811.2; *G.morhua* (*Gadus morhua*), ENSGMOG00000020190; *L.oculatus* (*Lepisosteus oculatus*), ENSLOCG00000017493; *A.mexicanus* (*Astyanax mexicanus*), ENSAMXG00000025368; *G.gallus* (*Gallus gallus*), NP_989526.1; *H.sapiens* (*Homo sapiens*), NP_005625.2. The HMG box characteristic of Sox proteins are framed and the specificity amino acids in HMG box domain are indicated with asterisks. Gaps (-) are introduced in the sequences to optimize the alignment. The percentage identities of the Japanese flounder Sox3 to its homologues are represented at the end of the sequences.

Table S1. Search results using MatInspector program from genomatic software suite.

Matrix Family	Detailed Family Information	Matrix	Detailed Matrix Information	Matrix Sim	Anchor Position	Strand	Sequence
V$AP1F	AP1, Activating protein 1	V$AP1.01	Activator protein 1	0.964	44	−	aagtgACTCatta
V$MYT1	MYT1 C2HC zinc finger protein	V$MYT1L.01	Myelin transcription factor 1-like, neuronal C2HC zinc finger factor 1	0.986	168	−	ggaaAGTTtaatt
V$CAAT	CCAAT binding factors	V$NFY.04	Nuclear factor Y (Y-box binding factor)	0.928	289	+	ctctCCAAtccgact
V$HOMF	Homeodomain transcription factors	V$BSX.01	Brain specific homeobox	0.94	326	+	tattcaacAATTatagaat
V$SORY	SOX/SRY-sex/testis determinig and related HMG box factors	V$SOX5.01	Sox-5	0.986	329	+	attcaaCAATtatagaattgtct
V$STEM	Motif composed of binding sites for pluripotency or stem cell factors	V$OCT3_4.02	POU domain, class 5, transcription factor 1	0.924	446	−	ggtatgtGCATttctgcgc
V$STEM	Motif composed of binding sites for pluripotency or stem cell factors	V$OCT3_4.02	POU domain, class 5, transcription factor 1	0.919	615	−	gccatttGCATgtgtcctt
V$NEUR	NeuroD, Beta2, HLH domain	V$NEUROG.01	Neurogenin 1 and 3 (ngn1/3) binding sites	0.904	620	−	cagCCATttgcat
V$SP1F	GC-Box factors SP1/GC	V$SP1.03	Stimulating protein 1, ubiquitous zinc finger transcription factor	0.924	794	−	tggagGGGCcgggccat
V$AP1F	AP1, Activating protein 1	V$AP1.01	Activator protein 1	1	947	−	gaatgAGTCagac
V$HOMF	Homeodomain transcription factors	V$BSX.01	Brain specific homeobox	0.905	970	−	atgttgtgAATTgtcttta
V$HOMF	Homeodomain transcription factors	V$BSX.01	Brain specific homeobox	0.975	1049	+	ttactggtAATTataacta
V$CREB	cAMP-responsive element binding proteins	V$CREB1.02	cAMP-responsive element binding protein 1	0.955	1066	−	aaaagATGAagtcatttatag
V$MYT1	MYT1 C2HC zinc finger protein	V$MYT1L.02	MyT1 zinc finger transcription factor involved in primary neurogenesis	0.985	1108	+	taaAAGTttggtc
V$HOXC	HOX–PBX complexes	V$MEIS1.03	Meis homeobox 1	0.984	1133	−	tgcatGATTtattttta
V$MYT1	MYT1 C2HC zinc finger protein	V$MYT1L.02	MyT1 zinc finger transcription factor involved in primary neurogenesis	1	1228	+	aaaAAGTttttaa
V$TALE	TALE homeodomain class recognizing TG motifs	V$TGIF.01	TG-interacting factor belonging to TALE class of homeodomain factors	1	1285	+	ctgttttatGTCAaagt
V$TALE	TALE homeodomain class recognizing TG motifs	V$TGIF.01	TG-interacting factor belonging to TALE class of homeodomain factors	1	1361	−	ccacctgttGTCAaaca
V$HOXC	HOX–PBX complexes	V$MEIS1.03	Meis homeobox 1	0.986	1382	+	taaatGATTtattttct
V$HOMF	Homeodomain transcription factors	V$BSX.01	Brain specific homeobox	0.957	1487	+	gcctctgtAATTagactaa
V$TALE	TALE homeodomain class recognizing TG motifs	V$TGIF.01	TG-interacting factor belonging to TALE class of homeodomain factors	1	1506	−	aggatcaatGTCAgtaa

Table S1. *Cont.*

Matrix Family	Detailed Family Information	Matrix	Detailed Matrix Information	Matrix Sim	Anchor Position	Strand	Sequence
V$SORY	SOX/SRY-sex/testis determinig and related HMG box factors	V$SOX3.01	SRY-box containing gene 3	0.95	1685	−	ctgcaaCAAAtgaaaagcaaagc
V$SORY	SOX/SRY-sex/testis determinig and related HMG box factors	V$SOX3.01	SRY-box containing gene 3	0.979	1733	+	cagaaaCAAAagcctcttgtagt
V$SORY	SOX/SRY-sex/testis determinig and related HMG box factors	V$SOX6.01	SRY (sex determining region Y)-box 6	0.939	1764	−	atctgACAAtgaagacgagcagt
V$TALE	TALE homeodomain class recognizing TG motifs	V$TGIF.01	TG-interacting factor belonging to TALE class of homeodomain factors	1	1768	+	gtcttcattGTCAgata
V$MYT1	MYT1 C2HC zinc finger protein	V$MYT1L.02	MyT1 zinc finger transcription factor involved in primary neurogenesis	0.992	1835	−	gtaAAGTtttaaa
V$CAAT	CCAAT binding factors	V$NFY.01	Nuclear factor Y (Y-box binding factor)	0.987	1914	−	gcgaCCAAtcagcgc
V$SORY	SOX/SRY-sex/testis determinig and related HMG box factors	V$SOX6.01	SRY (sex determining region Y)-box 6	0.981	1929	−	gcacgACAAagagcagccggcga
V$TALE	TALE homeodomain class recognizing TG motifs	V$TGIF.01	TG-interacting factor belonging to TALE class of homeodomain factors	1	1941	+	tgtcgtgctGTCAgtcc
V$PBXC	PBX - MEIS complexes	V$PBX3.01	Pre-B-cell leukemia homeobox 3	0.974	1945	−	atgtggacTGACagcac
V$EBOX	E-box binding factors	V$USF.04	Upstream stimulating factor 1/2	0.903	1957	+	cacatCACAtggccact
V$NEUR	NeuroD, Beta2, HLH domain	V$NEUROG.01	Neurogenin 1 and 3 (ngn1/3) binding sites	0.927	1957	−	tggCCATgtgatg
V$MYT1	MYT1 C2HC zinc finger protein	V$MYT1L.02	MyT1 zinc finger transcription factor involved in primary neurogenesis	0.902	2095	−	aaaAAGTtctcgc
V$TALE	TALE homeodomain class recognizing TG motifs	V$TGIF.01	TG-interacting factor belonging to TALE class of homeodomain factors	0.998	2159	+	cgctgttttGTCAactt
V$AP1F	AP1, Activating protein 1	V$AP1.03	Activator protein 1	0.905	2187	+	ctgTGAGtgaaca

Solution parameters equence files: Posox3.seq(1 sequences, 2208 bp); **Family matches**: yes; **MatInspector library**: Matrix Family Library Version 9.2 (October 2014); **Selected groups**: General Core Promoter Elements (0.90/Optimized) (core/matrix sim): Vertebrates (0.90/Optimized).
